# A benchmark for assessing large language models on molecular-to-food and food-to-molecular prediction tasks

**DOI:** 10.3389/frai.2026.1818923

**Published:** 2026-07-10

**Authors:** Jin Pan, Xinglin Pan, Hao Pan

**Affiliations:** 1 School of Economics and Management, Yango University, Fuzhou, China; 2 The Hong Kong University of Science and Technology (Guangzhou), Guangzhou, China

**Keywords:** artificial intelligence in food science, benchmark, hallucination, large language models, molecular-to-food mapping

## Abstract

Large Language Models (LLMs) have demonstrated remarkable proficiency in general-purpose tasks, yet their capacity for fine-grained reasoning in knowledge-intensive domains (KIDs) remains largely unexplored. This study addresses this gap by investigating LLM performance in the specialized field of food chemistry. We introduce a novel benchmark comprising two core tasks: Molecular-to-Food Prediction (MFP) and Food-to-Molecular Prediction (FMP). To support this benchmark, we curated and standardized the FlavorDB dataset, creating a robust testbed for flavor-molecular association. We evaluated two open-source models (Kimi-K2, DeepSeek-V3.2) and three closed-source models (Gemini-3-Pro, GPT-5.1, and Seed-1.8) under zero-shot and one-shot in-context learning settings. Our systematic analysis yields six key findings that characterize the capabilities and limitations of current LLMs in this domain. For instance, in the FMP task, Gemini-3-Pro achieved the highest zero-shot F1 score of 0.556, while Kimi-K2 led the one-shot setting with an F1 score of 0.522. In-context learning consistently improved performance across models, most notably boosting Kimi-K2's F1 score from 0.451 to 0.496 on complex multi-food tasks. Critically, we identify four recurring categories of domain-specific reasoning errors, which illuminate the fundamental challenges general-purpose models face when applied to fine-grained scientific inference in food chemistry. This work not only establishes a framework for rigorously evaluating LLM potential in knowledge-intensive domains but also provides a critical foundation for advancing practical applications, including flavor optimization and the development of novel food products.

## 1 Introduction

Large Language Models (LLMs) show high proficiency in general tasks like reasoning and question answering (Brown et al., 2020; DeepSeek-AI, 2025; Wei et al., 2022). For instance, Claude-Sonnet-4.6 model achieves a score of 89.3% on the MMMLU (Hendrycks et al., 2021) benchmark, highlighting its broad multitask language understanding capabilities. However, their reliability in Knowledge-Intensive Domains (KIDs) remains a subject of critical debate (Ji et al., 2023; Zhong et al., 2024). KIDs are defined by high knowledge density and fine-grained semantic constraints. In these fields, accurate inference requires more than surface-level pattern matching; it requires the ability to map complex relations within specialized data systems. Evaluating LLMs in KIDs therefore requires testing whether they can integrate fragmented factual data into precise, discriminative reasoning.

Chemistry is a prime example of a KID, and recent literature has begun to map LLM capabilities in this space. For instance, (White et al. 2023) introduced an expandable framework for evaluating chemistry knowledge by prompting models to solve problems as coding tasks. Similarly, (Runcie et al. 2026) developed ChemIQ, a benchmark of 796 questions focused on molecular comprehension and organic chemistry reasoning.

Within the food science domain, deep learning and LLM-based approaches have begun to assist in identifying bioactive peptides (Geng et al., 2025; Qin et al., 2024; Jiang et al., 2022), monitoring food quality (Lun et al., 2025; Class et al., 2021), and optimizing extrusion processing parameters (Bölücü et al., 2025). Despite these advancements, a critical research gap persists: there is currently no standardized framework to systematically evaluate the depth of LLMs' knowledge regarding the bidirectional relationship between molecules and food. This lack of a benchmark hinders our understanding of the boundaries and limitations of general-purpose AI when applied to fine-grained food chemistry reasoning (Huang et al., 2025; Osinomumu et al., 2025). While these existing studies address specific industrial optimizations, we focus on the more fundamental and specialized bidirectional mapping between microscopic molecular structures and macroscopic food sources. This relationship is inherently difficult to model: a single food source can contain hundreds of compounds, while a specific molecule may appear in taxonomically unrelated species. This complexity raises a fundamental research question: *Can LLMs accurately navigate these bidirectional molecular-food mappings?*

To address this gap, we establish a comprehensive evaluation framework consisting of two core tasks: Molecular-to-Food Prediction (MFP) and Food-to-Molecular Prediction (FMP). Using a curated version of the FlavorDB dataset (Garg et al., 2018), we evaluated the performance of leading open-source models [e.g., Kimi-K2 (Kimi-Team, 2025) and DeepSeek-V3.2 (DeepSeek-AI, 2025)] and closed-source models [e.g., Gemini-3-Pro (DeepMind, 2025) and GPT-5.1 (OpenAI, 2025)] under zero-shot and one-shot in-context learning settings (Brown et al., 2020).

Our systematic analysis yields six key findings that characterize the current state of LLMs in flavor-molecular associations, highlighting the effectiveness of in-context learning and the competitive performance of certain open-source architectures. Furthermore, through targeted case studies, we identify and categorize specific reasoning errors, such as hallucinated processing assumptions and over-reliance on general biological principles, that LLMs exhibit in this domain (Huang et al., 2025). By systematically mapping both successes and failures, this work provides a critical foundation for advancing practical applications in food science, like flavor optimization, while also charting a path for the development of future domain-specific models.

## 2 Related work

### 2.1 Hallucinations and benchmarking

Hallucinations—outputs that are fluent but factually incorrect—pose a significant risk in scientific domains. (Anh-Hoang et al. 2025) attribute these errors to a combination of model internals and prompt sensitivity. In specialized fields, these errors often stem from outdated training data or the inclusion of information not present in source documents (Freire et al., 2024). Such failures are particularly problematic in engineering or medical settings, where accuracy is mandatory (Ghosh and Mittal, 2025). To mitigate these risks, (Vilakati 2025) shows that structured prompt engineering, such as stepwise instructions, can serve as a safeguard against reasoning errors.

Detecting these hallucinations requires rigorous benchmarking tailored to specific domains rather than general language tasks (Polonioli, 2025). Recent efforts have produced benchmarks for clinical healthcare (Moëll et al., 2025) and manufacturing documentation (Freire et al., 2024). These studies emphasize that benchmarks must measure factual grounding and robustness within a specific context to be useful for scientific workflows.

### 2.2 LLMs for knowledge-intensive domains

By leveraging Retrieval-Augmented Generation (RAG) frameworks and instruction-based fine-tuning, LLMs have emerged as robust tools for information retrieval and expert-level reasoning across knowledge-intensive domains (Randolph et al., 2025; Freire et al., 2024). Unlike traditional retrieval systems, LLM-RAG implementations in closed-library scenarios enable precise question-answering and automated assessment generation within specialized fields like manufacturing and pharmacy education, where they frequently outperform open-source alternatives in streamlining complex workflows (Freire et al., 2024; Yang et al., 2024). In pharmaceutical and clinical contexts, models demonstrate significant potential for drug-drug interaction prediction and diagnostic support, e.g., spoken language depression recognition—by integrating domain-specific psychological expertise and in-context judging frameworks (Qi et al., 2025; Li et al., 2025b). Furthermore, the synergy between LLMs and knowledge graphs addresses inherent hallucination risks, facilitating collaborative ontology engineering and secure, federated synchronization of semantic knowledge bases (Kampars et al., 2025; Cai et al., 2025; Li et al., 2025a). These advancements establish a scalable foundation for cross-disciplinary knowledge management, ensuring that generative capabilities remain grounded in the rigorous legal and technical requirements of high-stakes professional environments (Knoedler et al., 2024; Ghanem and Cruz, 2025).

### 2.3 LLMs for molecular property prediction

Through instruction tuning on large-scale chemical corpora and SMILES molecular structures, large language models have become efficient generalizable tools in the field of Molecular Property Prediction (MPP), demonstrating inferential potential that surpasses traditional task-specific models (Weininger, 1988; Weininger et al., 1989; Zhou et al., 2025; Zhuang et al., 2025). Different from Graph Neural Networks (GNNs), models such as MolecularGPT transform molecular property prediction into an In-Context Learning (ICL) problem (Liu et al., 2024; Radford et al., 2019; Brown et al., 2020). They realize the alignment of chemical structural patterns and semantic knowledge by aggregating diverse task instructions, and can complete robust zero-shot inference for unseen tasks without retraining for a single task. Their prediction accuracy is comparable to that of dedicated graph-based architectures, laying a technical foundation for the application of large language models in molecular-to-food mapping reasoning in food chemistry.

This study uses this mapping task to determine whether LLMs have genuine chemical common sense: the ability to reason beyond word-vector co-occurrence and grasp the fundamental relationships between molecules and complex biological matrices. This limitation is the key barrier preventing current general-purpose large models from successful application to knowledge-intensive fields.

## 3 Task and data

In this section, we define specific evaluation benchmarks and datasets to test how accurately LLMs can identify food items from complex molecular flavor profiles. Rather than simple pattern matching, these tasks are designed to challenge the model's chemical reasoning, specifically its ability to interpret molecular data within the biological and culinary context of food science.

### 3.1 Task definition

#### 3.1.1 Molecular-to-food prediction

The MFP task evaluates whether the LLM can identify all potential food sources that share a common set of molecular markers. Formally, given a set of input molecules *M* = {*m*_1_, *m*_2_, …, *m*_*n*_}, the model acts as a mapping function *h*(*M*) to predict a subset of foods *F* = {*f*_1_, *f*_2_, …, *f*_*m*_} that contain these specific compounds. This task simulates a “reverse search” scenario in food chemistry: if a lab detects a specific volatile profile, can the generative AI list all possible ingredients (e.g., various citrus fruits or fermented products) that could have produced that profile? This tests the model's associative memory regarding chemical-food co-occurrence. We instantiate three sub-tasks of increasing difficulty by varying the size of the input molecule set: |*M*| = 1 (single molecule), |*M*| = 2 (two molecules), and |*M*| = 3 (three molecules). Larger |*M*| imposes stricter joint-constraints and tests the model's ability to identify foods that simultaneously contain all specified molecules.

#### 3.1.2 Food-to-molecular prediction

In contrast, the FMP task requires the model to reconstruct the characteristic molecular “fingerprint” of a specific food item. Here, the mapping *g*(*F*) → *M* assesses the model's generative precision. This task requires the LLM to distinguish between closely related species (e.g., distinguishing a lemon from a lime based on subtle concentration differences in citral and limonene). By framing the task this way, we evaluate if LLMs can perform discriminative chemical reasoning comparable to a trained flavorist or sensory scientist, moving beyond simple data recall to understand the nuances of food composition. Correspondingly, we define three FMP sub-tasks with |*F*| = 1 (single food item), |*F*| = 2 (two food items), and |*F*| = 3 (three food items), where larger |*F*| requires the model to synthesize and reconstruct the union of molecular profiles across multiple foods simultaneously.

### 3.2 Data and analysis

#### 3.2.1 Dataset

Our analysis builds upon FlavorDB,^1^ which we process following the methodology of FoodPuzzle (Huang et al., 2025). The original data undergoes a standardized cleaning and structuring procedure, yielding three core components. The first component profiles individual flavor molecules, detailing their chemical properties and sensory characteristics. The second component catalogs food items, including their category and molecular composition. The third component is a binary association matrix that explicitly maps each food item to its constituent flavor molecules. This structured representation enables a comprehensive investigation into the relationship between molecular features and culinary identity. The dataset encompasses a wide range of food categories; their distribution is summarized in Table 1.

**Table 1:** Distribution of food categories in the processed dataset.

Categories and counts					
Fish	Fruit	Dish	Herb	Dairy	Meat
120	81	77	51	48	47
Plant	Alcoholic beverage	Berry	Essential oil	Seafood	Bakery
45	44	42	42	35	32
Spice	Additive	Vegetable	Cereal	Legume	Nut
25	25	24	23	23	21
Plant derivative	Cabbage	Beverage	Fungus	Vegetable tuber	Citrus fruit
20	16	13	11	10	10
Flower	Vegetable root	Gourd	Caffeinated beverage	Maize	Seed
9	8	8	7	6	5
Vegetable fruit	Animal product	Berry fruit	Fruit essence	Vegetable stem	
4	1	1	1	1	

#### 3.2.2 Visualization analysis

A core challenge (and defining feature) of the molecular-to-food prediction task stems from a fundamental duality: while similar foods often share similar molecular profiles, it is the presence or absence of key distinguishing molecules that defines unique food identities. Therefore, a model's success hinges on its ability to discern general shared molecular patterns from specific discriminative signatures.

**Figure 1:**
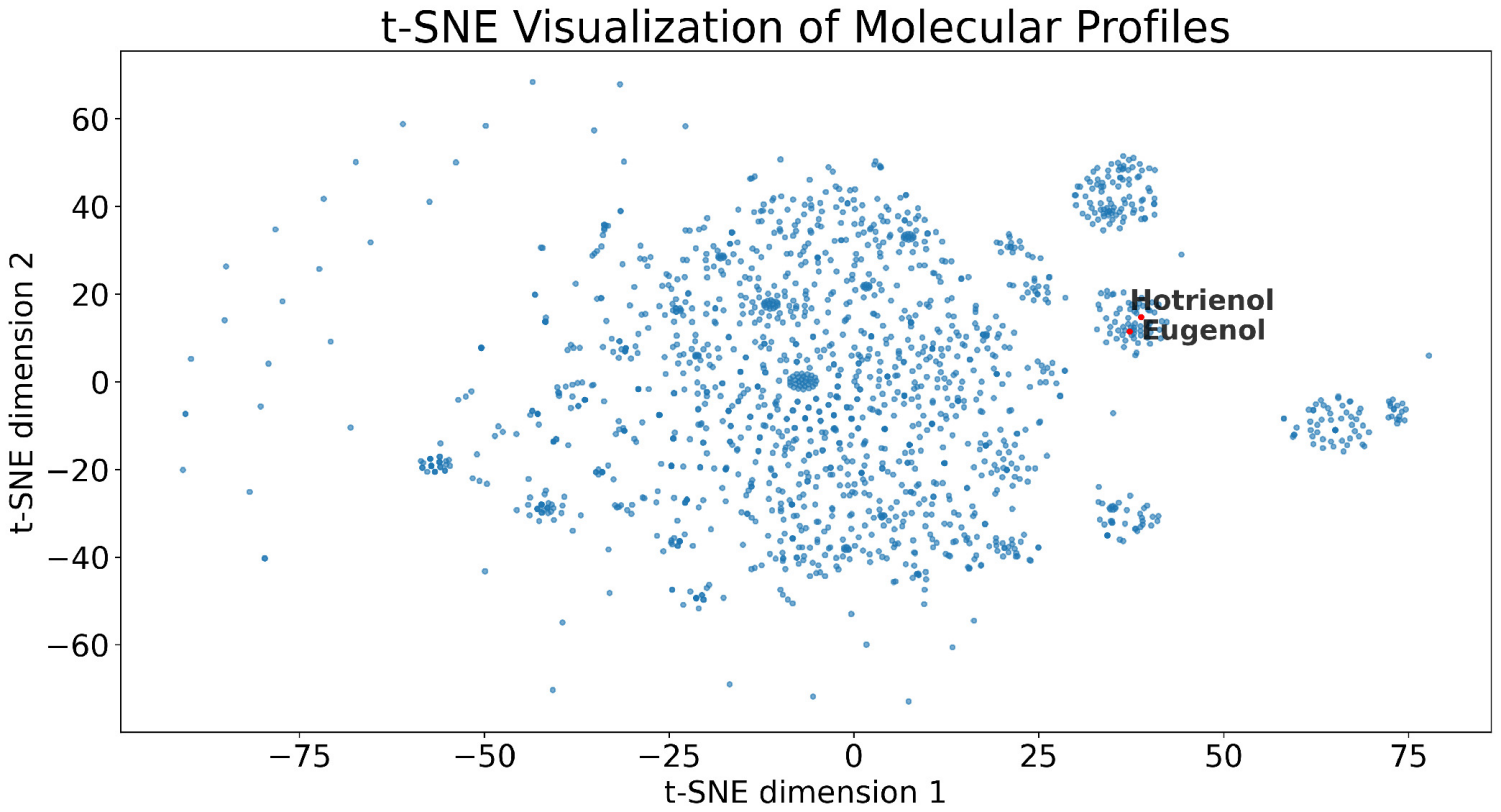
t-SNE projection of molecular embeddings. Each point represents a unique flavor molecule; points close in the embedding space indicate molecules that frequently co-occur across food items. The clustering of Eugenol and Hotrienol exemplifies the challenge in MFP: disparate chemical structures can occupy the same embedding region due to their sensorially-related co-occurrence patterns (here in wine varieties).

To facilitate this analysis, we construct embeddings of molecules and foods from the binary association matrix. Each molecule is represented as a binary occurrence vector across all foods in the dataset (1 if present, 0 otherwise); each food is similarly represented as a binary vector over its constituent molecules. To visualize these high-dimensional embeddings, we employ t-distributed Stochastic Neighbor Embedding (t-SNE) (Hinton and Roweis, 2002; Van der Maaten and Hinton, 2008), a nonlinear dimensionality-reduction technique that maps high-dimensional vectors into a low-dimensional (here, two-dimensional) space while preserving local neighborhood structure, such that points close together in the original space remain close in the projection. As shown in Figure 1, our analysis of the molecular embedding space reveals a compelling topological pattern: molecules with disparate chemical structures, such as *Eugenol* and *Hotrienol*, are projected into proximate regions within the t-SNE manifold. This proximity is driven by their frequent co-occurrence in a specific cluster of sensorially related beverages, including *Plum Wine, Port Wine*, and *Red Wine*. Such distributional overlap highlights a fundamental challenge in the MFP task: the requirement for models to disambiguate culinary entities that possess highly similar molecular fingerprints. Consequently, successful prediction necessitates more than rote memorization of molecule-food associations; it requires the model to decode the subtle, combinatorial logic that governs how specific molecular ensembles define unique food identities.

**Figure 2:**
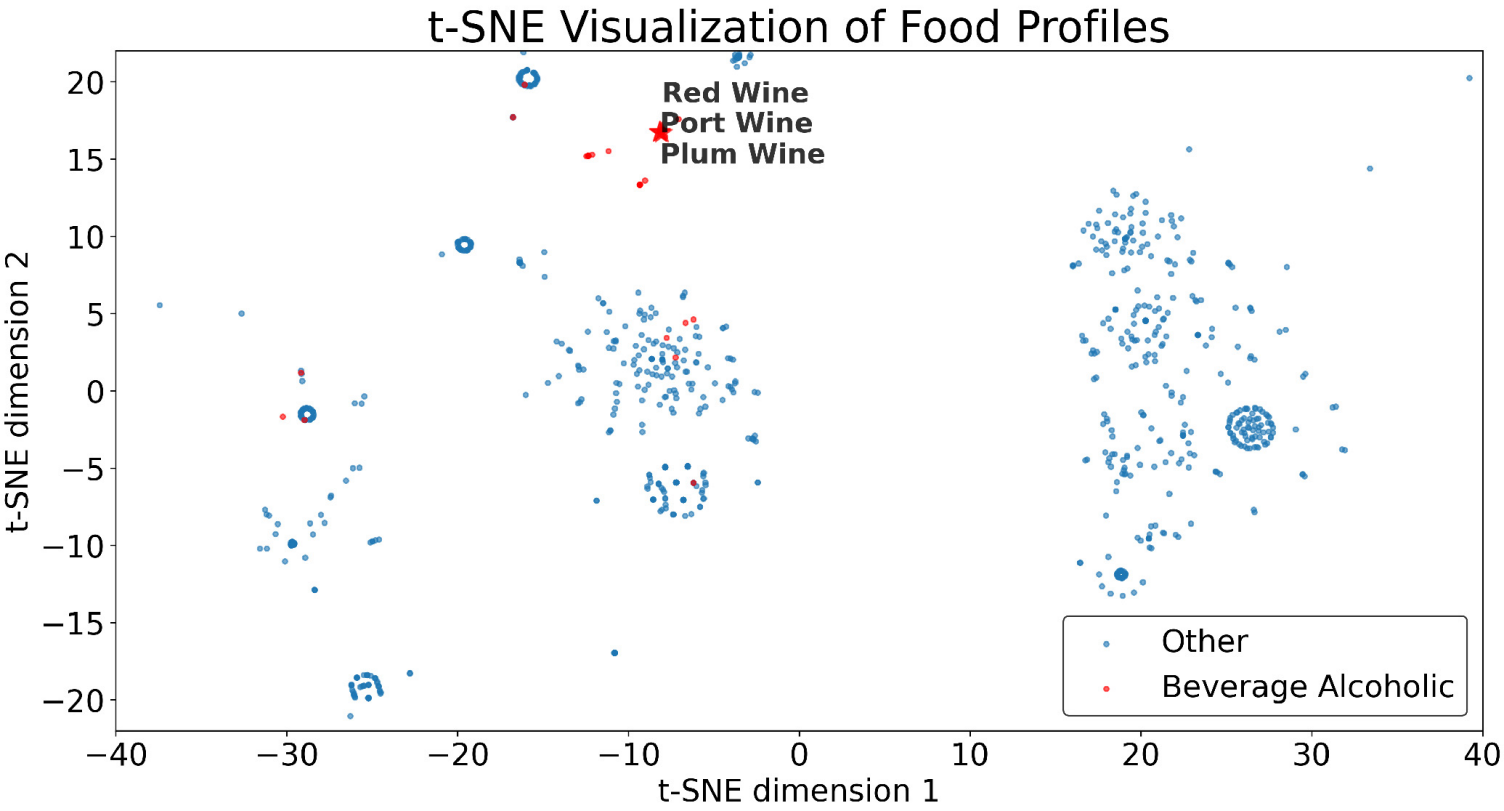
t-SNE projection of food embeddings derived from the binary food–molecule association matrix. Each point represents a food item; red points denote alcoholic beverages. The zoomed cluster (marked with star) highlights Red Wine, Port Wine, and Plum Wine in close proximity, driven by their shared molecular signatures. Notably, unique molecules such as Pyrrolidine appear exclusively in Red Wine, illustrating the precision required to distinguish foods with highly similar but not identical molecular profiles.

To validate the effect of this molecular co-occurrence on food categories, we conducted a parallel t-SNE visualization on food entity embeddings. As Figure 2 shows, alcoholic beverages including Red Wine, Port Wine, and Plum Wine form a cluster, corresponding directly to their shared molecular signatures. Notably, we also identify molecules unique to a single entity within this group, such as Pyrrolidine found exclusively in Red Wine. This precise interplay between shared and unique molecular features encapsulates the core challenge of the prediction problem.

### 3.3 Evaluation protocols

To quantify the alignment between the free-text outputs generated by LLMs and the ground truth labels, we employ a semantic-based evaluation framework. Given that models may produce semantically equivalent but syntactically distinct strings (e.g., *Millet Beer* vs. *Bantu Beer*), a strict string matching approach would underestimate model performance. To this end, we define a semantic indicator function *I*_*sem*_(*a, b*)∈{0, 1}, which evaluates to 1 if an auxiliary language model determines *a* and *b* are semantically equivalent, and 0 otherwise.

To validate the reliability of the semantic equivalence judgment, we randomly sampled 50 predicted–ground–truth pairs and subjected them to independent expert annotation. The auxiliary LLM's assessments achieved perfect agreement with the human expert. This near-trivial matching outcome is a direct consequence of our task design: the prompt explicitly lists the candidate food items (or molecules), and models overwhelmingly reproduce those exact candidate names in their output, with variations limited to minor differences in capitalization, whitespace, or delimiter formatting.

#### 3.3.1 Evaluation of MFP

For the Molecular-to-Food Prediction task, let $\hat{F}$ be the set of predicted food items and F^*^ be the set of ground truth items. The performance is assessed using the *F*_1_ score, which balances precision *P* and recall *R*. Precision is defined as the proportion of predicted items that find a semantic match in the ground truth:


$$\begin{array}{l} P=\frac{1}{\left|\hat{F}\right|}\underset{\hat{f}\in \hat{F}}{\sum }\underset{f^{*}\in F^{*}}{\max}I_{sem}\left(\hat{f},f^{*}\right) \end{array}$$ (1)

Similarly, recall measures the coverage of ground truth items by the predictions:


$$\begin{array}{l} R=\frac{1}{\left|F^{*}\right|}\underset{f^{*}\in F^{*}}{\sum }\underset{\hat{f}\in \hat{F}}{\max}I_{sem}\left(f^{*},\hat{f}\right) \end{array}$$ (2)

The final *F*_1_ score is computed as the harmonic mean: *F*_1_ = 2·(*P*·*R*)/(*P*+*R*).

In the MFP task, the *F*_1_ score serves as a comprehensive metric to determine how reliably an LLM can identify specific food items from a complex chemical fingerprint. ❶ If both *P* and *R* are high, it signifies that the LLM has a robust understanding of molecular signatures, accurately pinpointing the correct food without making irrelevant guesses. ❷ A high *R* but low *P* suggests the model is over-predicting; while it successfully captures the correct food item, it also generates numerous false positives (e.g., predicting multiple unrelated ingredients for a single flavor profile). ❸ Conversely, a high *P* but low *R* indicates a conservative model that is very accurate when it does speak, but it misses many valid food-molecule associations, failing to recognize the full scope of the ground truth. ❹ If both are low, the LLM fails to establish any meaningful link between the chemical compounds and the food matrix. Ultimately, the *F*_1_ score represents the overall predictive stability of the model, ensuring that the AI isn't just lucky or overly talkative, but truly precise in its biochemical mapping.

#### 3.3.2 Evaluation of FMP

The evaluation of the FMP task follows the same formal methodology as described for the MFP task. Performance is assessed using precision, recall, and their harmonic mean, the *F*_1_ score, based on semantic matching between the predicted set of molecules and the ground truth set.

The FMP task tests if the LLM can deconstruct a food item into its constituent flavor molecules. ❶ High *P* and *R* indicate that the LLM functions as an effective digital sommelier or chemist, correctly listing all the key volatile compounds that define a food's aroma. ❷ A low *P* paired with a high *R* means the LLM is hallucinating molecules; it manages to include the correct ones but litters the results with compounds that do not actually exist in that food's molecular profile. ❸ High *P* and low *R* signify that the model identifies genuine molecules but provides an incomplete profile, perhaps only recognizing the most common esters while ignoring secondary but essential terpenoids. ❹ Low scores in both metrics suggest the model lacks the domain-specific knowledge to bridge the gap between a culinary label and its chemical reality. In this context, the *F*_1_ score reflects the completeness and purity of the model's chemical reconstruction, which is vital for applications like synthetic flavor formulation or food quality control.

## 4 Evaluated methods

In this section, we delineate the selection of LLMs used as benchmarks. To provide a comprehensive assessment, we categorized the models into two distinct groups based on their accessibility and architectural transparency.

### 4.1 Open-source LLMs

In this study, we evaluate Kimi-K2 (Kimi-Team, 2025) and DeepSeek-V3.2 (DeepSeek-AI, 2025), two prominent open-source large language models. The primary advantage of utilizing open-source models is that they provide full transparency and reproducibility, which is crucial for validating novel analytical methods in food science. This allows other researchers to inspect, replicate, and build upon our work, thereby strengthening the reliability of the proposed molecular profiling framework.

Although these are general-purpose models not specifically trained for food science domains, we selected them for their proven capabilities in complex reasoning and long-context understanding (Kimi-Team, 2025; DeepSeek-AI, 2025), which are essential for interpreting intricate molecular data.

### 4.2 Closed-source LLMs

In addition to open-source models, we evaluate Seed-1.8 (Seed, 2026), GPT-5.1 (OpenAI, 2025), and Gemini-3-Pro (DeepMind, 2025), three leading closed-source large language models. The primary advantage of incorporating these models lies in their state-of-the-art performance and robust API ecosystems. These models typically undergo extensive reinforcement learning from human feedback (Ouyang et al., 2022) and are optimized for high-level reasoning, which provides a benchmark for the upper bounds of current AI capabilities in complex molecular interpretation.

While the internal architectures and training datasets of these models are not publicly disclosed, their superior cross-modal integration and massive context windows—particularly Gemini's ability to ingest entire molecular databases in a single prompt—make them indispensable for synthesizing multi-source food chemistry data. By comparing these proprietary models with open-source alternatives, we aim to assess whether the performance gains of leading-edge commercial AI justify the trade-off in transparency and computational cost for food science applications.

### 4.3 In-context learning

In-context learning (ICL) refers to the ability of large language models to perform a new task by conditioning on one or more demonstrations provided in the prompt, without any gradient updates or parameter modifications (Brown et al., 2020; Chen et al., 2022; Gu et al., 2023). Specifically, in the zero-shot setting, the model receives only the task instruction and the input (molecular profile or food item) with no worked example; in this paper we employ few-shot prompting with a single exemplar (one-shot), where a gold-standard reasoning example demonstrates the correct process before each test query. We further investigate in-context learning techniques to refine the models' task-specific execution. A significant challenge in food science is that specific molecule names often constitute out-of-distribution information for LLMs due to limited exposure during general pre-training, which can lead to hallucinations or impaired reasoning.

To mitigate this and provide a standardized reasoning framework, we utilized Gemini-3-Pro to generate a high-quality, step-by-step reasoning exemplar (using a distinct food-item case to avoid data leakage). This exemplar serves as a gold-standard reference in our few-shot prompts, enabling all other evaluated LLMs to simulate the sophisticated analytical logic and structured output format established by Gemini. This approach ensures that the performance variance across models stems from their inherent processing capabilities rather than inconsistencies in prompt interpretation. This strategy of using a strong model to generate a standardized reasoning exemplar aligns with prior work on in-context learning and chain-of-thought prompting (Brown et al., 2020; Wei et al., 2022; Chen et al., 2022; Gu et al., 2023).

## 5 Experiments

### 5.1 Experimental settings

We evaluated the performance of the candidate models using a test set of 200 randomly selected samples. We employed stratified random sampling to ensure the test set represents a diverse range of food categories and chemical structures. We implemented a sanity check to ensure that the one-shot exemplar was distinct from the test set in both food entities and molecular constituents, preventing intra-prompt data leakage. No model training or fine-tuning of any kind was conducted on any portion of the dataset, preserving the zero-shot and one-shot nature of the evaluation. To ensure reproducibility, structured prompt templates were designed for both tasks to guide the models' reasoning process, as shown in Table 2.

**Table 2:** Prompt templates used for the MFP task (left) and the FMP task (right).

(a) MFP prompt structure	(b) FMP prompt structure
**Role:** You are an expert Food Scientist specializing in Molecular Gastronomy.	**Role:** You are an expert Food Scientist specializing in Molecular Gastronomy.
**Task:** Identify which candidate foods contain EVERY molecule listed in the provided profile.	**Task:** Identify which candidate molecules are present in EVERY food item listed in the provided profile.
**Data input:** •*Molecular Profile:* [A list of detected molecules] •*Candidate Foods:* [A list of potential food items to be verified]	**Data Input:** •*Food Item(s):* [A list of food items] •*Candidate Molecules:* [A list of potential molecules to be verified]
**Instructions:** Please reason through the task step by step. After your reasoning, provide your final answer strictly in the following format:	**Instructions:** Please reason through the task step by step. After your reasoning, provide your final answer strictly in the following format:
#### < matched foods separated by comma>	#### < matched molecules separated by comma>
If no foods match, write: #### None	If no molecules match, write: #### None

Regarding the model hyperparameters, we employed the default recommended settings for temperature and *top_p*. The *max_tokens* parameter was set to the model's upper limit to prevent any truncation of the generated reasoning or final results.

While we cannot definitively determine whether the specific FlavorDB pairs appeared in the models' pre-training data, we mitigated leakage concerns by evaluating a diverse set of five models. The consistency of results across architectures, along with the recurrence of similar reasoning errors, indicates that the benchmark probes generative reasoning rather than rote retrieval of pre–trained information.

### 5.2 MFP results

The experimental results for the MFP task are summarized in Table 3. This task evaluates the models' ability to identify corresponding food items based on molecular profiles across three sub-tasks of increasing complexity (|*M*| = 1, 2, 3). In the single-molecule setting (|*M*| = 1), Gemini-3-Pro achieves the highest zero-shot F1 score of 0.474, while Kimi-K2 leads the one-shot setting with an F1 score of 0.502. Across all levels of complexity, Gemini-3-Pro consistently performs best in zero-shot scenarios, whereas Kimi-K2 demonstrates superior adaptation in one-shot settings.

**Table 3:** Performance comparison of LLMs on the MFP task under 0-shot and 1-shot settings (F1 Score, Mean ± Std).

subTask	Setting	Kimi-K2	DeepSeek-V3.2	Gemini-3-Pro	GPT-5.1	Seed
|*M*| = 1	0-shot	0.464 ± 0.377	0.324 ± 0.365	**0.474 ± 0.370**	0.112 ± 0.265	0.395 ± 0.378
	1-shot	**0.502 ± 0.366**	0.379 ± 0.378	-	0.232 ± 0.336	0.453 ± 0.371
|*M*| = 2	0-shot	0.421 ± 0.375	0.286 ± 0.366	**0.442 ± 0.377**	0.128 ± 0.278	0.289 ± 0.366
	1-shot	**0.467 ± 0.365**	0.380 ± 0.377	-	0.256 ± 0.357	0.345 ± 0.378
|*M*| = 3	0-shot	0.397 ± 0.399	0.256 ± 0.361	**0.398 ± 0.360**	0.115 ± 0.285	0.252 ± 0.350
	1-shot	**0.417 ± 0.392**	0.239 ± 0.344	-	0.200 ± 0.337	0.231 ± 0.353

Gemini-3-Pro 1-shot entries are blank due to self-sampling constraints. Bold values indicate the highest F1 score within each sub-task and setting across all evaluated models.

**Figure 3:**
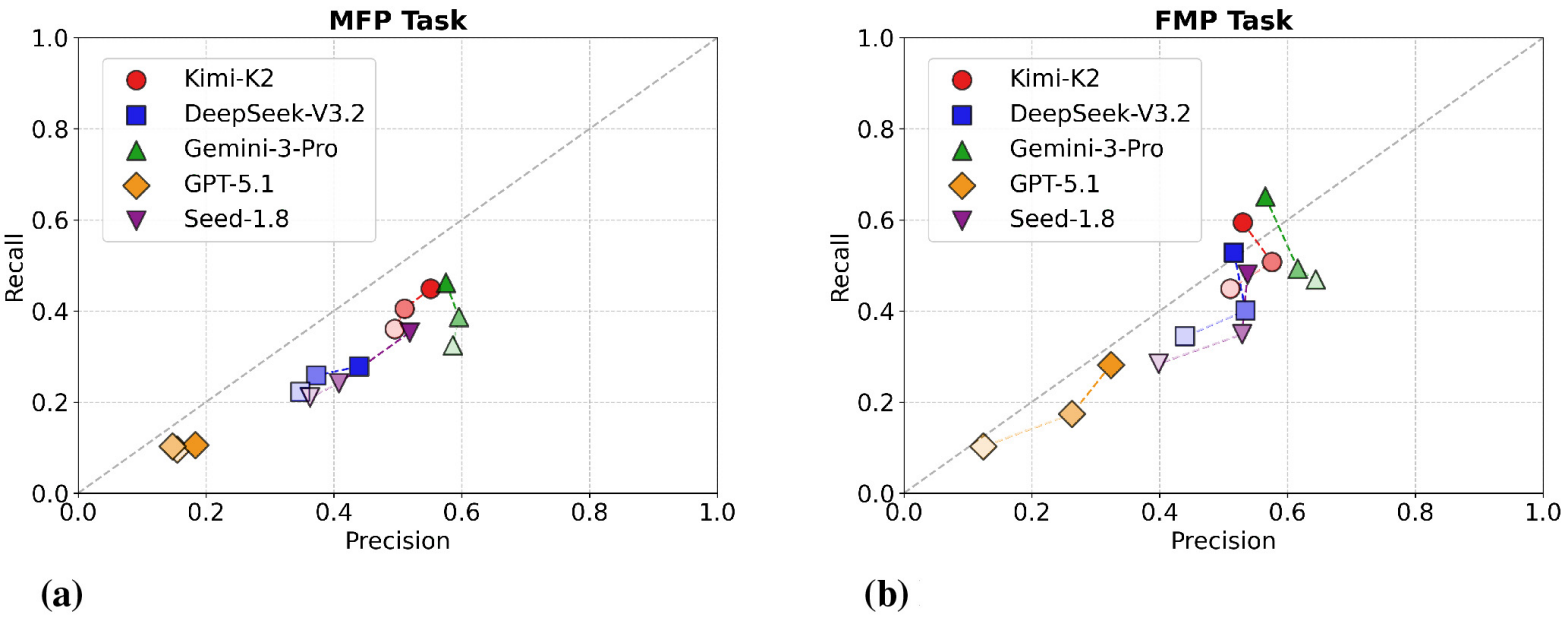
Precision–Recall distribution of the five evaluated LLMs across the two core prediction tasks. Each model is shown in a distinct color and marker. Within each model, darker shading indicates the simpler sub-task (|*M*| or |*F*| = 1) and lighter shading indicates the more complex sub-task (|*M*| or |*F*| = 3), reflecting increasing task difficulty. Points in the upper-right corner indicate jointly high precision and recall. The overall rightward position of (b) FMP compared to (a) MFP illustrates Insight 6 (generative reasoning outperforming retrieval-based association). **(a)** Molecular-to-food prediction (MFP). **(b)** Food-to-molecular prediction (FMP).

To further investigate the trade-off between precision and recall, we visualize the model performances in Figure 3a. Gemini-3-Pro exhibits the highest precision clusters, consistently positioned toward the right side of the plot, though its recall shows a slight decline as the number of input molecules increases. In contrast, Kimi-K2 maintains a more balanced trajectory near the diagonal, suggesting a stable harmonic mean between precision and recall.

### 5.3 FMP results

Food-to-Molecular Prediction (FMP). The experimental results for the FMP task are summarized in Table 4. We evaluate the F1 scores across three sub-tasks (|*F*| = 1, 2, 3), where models are required to reconstruct characteristic molecular fingerprints for given food items. Overall, Gemini-3-Pro and Kimi-K2 emerge as the most robust models for food-molecular matching. In the single-food task (|*F*| = 1), Gemini-3-Pro achieves the highest zero-shot F1 score of 0.556. Meanwhile, Kimi-K2 demonstrates superior performance in the one-shot setting, reaching an F1 score of 0.522. It is noteworthy that the standard deviations remain relatively consistent across all models.

**Table 4:** Performance comparison of LLMs on the FMP task under 0-shot and 1-shot settings (F1 Score, Mean ± Std).

subTask	Setting	Kimi-K2	DeepSeek-V3.2	Gemini-3-Pro	GPT-5.1	Seed
|*F*| = 1	0-shot	0.512 ± 0.350	0.485 ± 0.372	**0.556 ± 0.353**	0.274 ± 0.356	0.467 ± 0.373
	1-shot	**0.522 ± 0.348**	0.495 ± 0.356	-	0.365 ± 0.360	0.434 ± 0.360
|*F*| = 2	0-shot	**0.511 ± 0.375**	0.438 ± 0.395	0.511 ± 0.366	0.197 ± 0.334	0.402 ± 0.380
	1-shot	**0.493 ± 0.378**	0.472 ± 0.390	-	0.348 ± 0.375	0.388 ± 0.361
|*F*| = 3	0-shot	0.451 ± 0.421	0.368 ± 0.421	**0.503 ± 0.367**	0.107 ± 0.285	0.314 ± 0.400
	1-shot	**0.496 ± 0.407**	0.409 ± 0.416	-	0.303 ± 0.397	0.393 ± 0.404

Gemini-3-Pro 1-shot entries are blank due to self-sampling constraints. Bold values indicate the highest F1 score within each sub-task and setting across all evaluated models.

The P-R characteristics for the FMP task are illustrated in Figure 3b. Compared to the MFP task, most models move toward the upper-right quadrant, indicating that identifying molecular fingerprints is generally more intuitive for these LLMs than the reverse search. Gemini-3-Pro and Kimi-K2 again lead the cohort, with Gemini achieving superior recall in several sub-tasks.

### 5.4 Key findings

**Insight 1:** Competitive Biochemical Reasoning in Open-Source LLMs.

Leading open-source models, specifically Kimi-K2, demonstrate biochemical reasoning capabilities comparable to those of state-of-the-art closed-source models. This offers an important insight for the food science research community: we can build autonomous, customizable, and interpretable AI-powered food analysis platforms based on robust open-source foundation models, rather than relying on expensive, opaque commercial APIs.

**Insight 2:** In-Context Learning Effectively Bridges Domain Gaps.

Transitioning from 0-shot to 1-shot settings yielded consistent performance gains across most models. For instance, Kimi-K2's F1 score rose from 0.451 to 0.496 in complex tasks (|*F*| = 3). For food scientists, this implies that providing even a single detailed example can significantly stabilize the model's predictive accuracy.

**Insight 3:** Performance Degradation Under High Structural Complexity.

An inverse relationship exists between the number of input variables (|*M*| or |*F*|) and predictive success. As the molecular profile grows, the reasoning load increases, indicating that current LLMs still struggle with the high-dimensional combinatorial logic required for complex food matrices.

**Insight 4:** High-Precision, Low-Recall: A Conservative Reasoning Pattern.

Most models exhibit higher precision than recall, meaning they are conservative predictors: they are likely to be correct when they identify a match, but they often fail to capture the full spectrum of possible food-molecule associations.

**Insight 5:** Concurrent Performance Decay in Precision and Recall.

As task complexity increases (from |*M*|, |*F*| = 1 to 3), we observe a simultaneous decline in both precision and recall across nearly all models. This indicates that increasing the number of molecular or food variables does not introduce a trade-off between precision and recall; rather, it systematically erodes the models' overall discriminative power. For food science applications, this reveals a fundamental limitation of LLMs when processing complex relationships between molecules and foods.

**Insight 6:** Generative Reasoning Outperforms Retrieval-Based Association in Bidirectional Mapping.

A fundamental asymmetry emerges between the two reasoning directions. The FMP task consistently yields higher F1 scores than the MFP task.

We interpret this through the lens of task structure. FMP represents *generative reasoning*: given a food item, the model unfolds it into associated chemical features. This direction is native to autoregressive training (Allen-Zhu and Li, 2025). MFP, by contrast, requires *retrieval-based associative reasoning*: given a molecular profile, the model must recover all foods that contain it. This is the inverse direction. Prior work shows that LLMs systematically fail at such reversal tasks, even when the forward direction is well-learned (Berglund et al., 2024).

This gap is therefore not surprising. LLMs are more reliable when reasoning outward from a known entity than when searching inward from abstract features. We expect this limitation to extend beyond food chemistry to other knowledge-intensive domains.

### 5.5 Case study

We present a detailed analysis of specific instances where the model failed to accurately predict the chemical constituents of various food items.

#### 5.5.1 Error type 1: incorrect assumption of food processing

The first category of error involves the incorrect assumption that certain molecules only exist in food as a result of specific cooking or thermal processing treatments. For example, the model committed a false positive by including *Oriental wheat*, incorrectly assuming the sample was roasted or processed to generate Maillard reaction products like *2,3-dimethylpyrazine*. Conversely, it committed a false negative by excluding *Shrimp*, failing to recognize that this same molecule is a documented volatile component often arising from standard drying or preparation methods. These errors demonstrate a failure to account for the actual state of the food data provided, favoring an assumed processing history instead.

#### 5.5.2 Error type 2: reliance on general biological principles

Another significant reasoning flaw is the reliance on general biological principles rather than specific chemical profiling data. This was evidenced when the model included *Geranium* as a source of *cis-3-Hexenal* (a false positive) based on the broad botanical heuristic that “green leaves contain Green Leaf Volatiles.” While biologically plausible in a general sense, the model failed to verify whether this specific molecule was actually present in the documented chemical profile of the ingredient. Such reliance on high-level biological assumptions leads the model to overlook the unique chemical diversity found across different plant species.

#### 5.5.3 Error type 3: erroneous generalization of genus-level profiles

Furthermore, the model frequently fails by erroneously generalizing the phytochemical profile of an entire genus to a specific, less common species. In the case of *Elliott's blueberry* (*Vaccinium elliottii*), the model hallucinated the presence of *Isoquercitrin* because the molecule is commonly found in other members of the *Vaccinium* genus, such as standard *common blueberries*. Despite a lack of evidence in the ground truth for this specific species, the model assumed chemical uniformity across the genus. This highlights a critical need for species-level precision in molecular prediction rather than relying on genus-wide generalizations.

#### 5.5.4 Error type 4: misapplication of flavor heuristics

Finally, the model often commits a flavor or scent logic error by assuming that a food's primary sensory profile dictates its entire molecular composition. For instance, the model incorrectly excluded *Green Beans* as a source of *2-Acetylpyridine* because *Green Beans* do not typically exhibit a “roasted” or “popcorn-like” aroma. Similarly, it reasoned that the “brown, sweet” flavor of *Sapodilla* would preclude the presence of “green” molecules like *cis-3-Hexenal*. In both cases, the model prioritized sensory heuristics over chemical reality, failing to recognize that trace volatiles often exist in foods despite not defining their primary flavor or aroma.

### 5.6 Error mechanism and attribution analysis

To move beyond a purely descriptive account of model failures, we conducted a quantitative attribution analysis of 100 identified error instances (50 from closed-source models and 50 from open-source models). We mapped our four empirical error types to four fundamental cognitive limitations: (1) lack of domain knowledge, (2) generalization bias, (3) semantic confusion, and (4) context length constraints.

Table 5 reveals a high degree of distributional consistency between open-source and closed-source models, indicating that both architectures encounter shared systemic challenges when performing fine-grained biochemical reasoning. We attribute Type 1 errors to a lack of domain knowledge, as models frequently fail to retrieve or apply the specific thermochemical transformations required for accurate prediction. Failures in Type 2 and Type 3 errors are mapped to generalization bias, where models prioritize broad taxonomic rules and coarse-grained category associations over the unique structural nuances of specific molecules. Type 4 errors are attributed to semantic confusion, stemming from a reliance on linguistic pattern matching within chemical names rather than the structural logic encoded in SMILES strings. Finally, a marginal subset of failures (1 of 100) is attributed to context length constraints, manifesting as attention dispersion and a loss of logical coherence during complex, multi-hop reasoning tasks.

**Table 5:** Mapping of error types to root mechanisms, with distribution across models.

Error type	Root mechanism	Closed-source	Open-source
Food processing	Lack of domain knowledge	10	8
Biological principles	Generalization bias	30	32
Genus generalization	Generalization bias	4	6
Flavor heuristics	Semantic confusion	5	4
-	Context length constraints	1	0

## 6 Conclusions

In this study, we evaluated the performance of LLMs in bidirectional molecular-to-food mapping. We established two core evaluation tasks: Molecular-to-Food Prediction and Food-to-Molecular Prediction, and constructed a standardized benchmark based on the curated FlavorDB dataset. This framework was used to assess two open-source models (Kimi-K2 and DeepSeek-V3.2) and three closed-source models (Gemini-3-Pro, GPT-5.1, and Seed-1.8) under zero-shot and one-shot in-context learning settings. Through systematic analysis, we summarized six key findings regarding the current capabilities and behavioral patterns of LLMs in processing these tasks. Furthermore, four typical domain-specific reasoning errors were identified, clarifying the fundamental limitations of general-purpose LLMs in fine-grained food chemistry inference. These insights provide a critical foundation for future research, which will focus on domain-specific instruction tuning to mitigate reasoning hallucinations.

From a practical standpoint, the experimental results delineate clear boundaries for the industrial deployment of general–purpose LLMs in food chemistry. Given the sub–0.6 F1 scores, the conservative prediction pattern, and the risk of hallucinated assumptions, any real–world application should involve a human verification step, particularly for safety–related tasks. Meeting these constraints could eventually lead to the safe integration of LLM–driven tools into industrial flavor science, bridging the gap from experimental validation to practical deployment.

## Data Availability

The datasets presented in this study can be found in online repositories. The names of the repository/repositories and accession number(s) can be found in the article/supplementary material.

## Footnotes

1
https://cosylab.iiitd.edu.in/flavordb

## References

[B1] Allen-Zhu Z. Li Y. (2025). “Physics of language models: Part 3.2, knowledge manipulation,” in ICLR (OpenReview.net). doi: 10.2139/ssrn.5250621

[B2] Anh-Hoang D. Tran V. Nguyen L. (2025). Survey and analysis of hallucinations in large language models: attribution to prompting strategies or model behavior. Front. Artif. Intell. 8:1622292. doi: 10.3389/frai.2025.1622292 41098969 PMC12518350

[B3] Berglund L. Tong M. Kaufmann M. Balesni M. Stickland A. C. Korbak T. . (2024). “The reversal curse: Llms trained on “a is b" fail to learn "b is a",” in ICLR (OpenReview.net).

[B4] Bölücü N. Pennells J. Yang H. Rybinski M. Wan S. (2025). An evaluation of large language models for supplementing a food extrusion dataset. Foods 14:1355. doi: 10.3390/foods14081355 40282757 PMC12026441

[B5] Brown T. B. Mann B. Ryder N. Subbiah M. Kaplan J. Dhariwal P. . (2020). “Language models are few-shot learners,” in NeurIPS.

[B6] Cai L. Yu C. Kang Y. Fu Y. Zhang H. Zhao Y. (2025). Practices, opportunities and challenges in the fusion of knowledge graphs and large language models. Front. Comput. Sci. 7:1590632. doi: 10.3389/fcomp.2025.1590632

[B7] Chen M. Du J. Pasunuru R. Mihaylov T. Iyer S. Stoyanov V. . (2022). “Improving in-context few-shot learning via self-supervised training,” in NAACL-HLT (Association for Computational Linguistics), 3558–3573. doi: 10.18653/v1/2022.naacl-main.260

[B8] Class L.-C. Kuhnen G. Rohn S. Kuballa J. (2021). Diving deep into the data: a review of deep learning approaches and potential applications in foodomics. Foods 10:1803. doi: 10.3390/foods10081803 34441579 PMC8392494

[B9] DeepMind G. (2025). Gemini 3 pro. Technical report, Google DeepMind.

[B10] DeepSeek-A I. (2025). Deepseek-v3.2: pushing the frontier of open large language models. CoRR, abs/2512.02556.

[B11] Freire S. K. Wang C. Foosherian M. Wellsandt S. Ruiz-Arenas S. Niforatos E. (2024). Knowledge sharing in manufacturing using llm-powered tools: user study and model benchmarking. Frontiers Artif. Intell. 7:1293084. doi: 10.3389/frai.2024.1293084 38601111 PMC11004332

[B12] Garg N. Sethupathy A. Tuwani R. Nk R. Dokania S. Iyer A. . (2018). Flavordb: a database of flavor molecules. Nucleic Acids Res. 46, D1210–D1216. doi: 10.1093/nar/gkx957 29059383 PMC5753196

[B13] Geng H. Xu C. Ma H. Dai Y. Jiang Z. Yang M. . (2025). In silico discovery and sensory validation of umami peptides in fermented sausages: a study integrating deep learning and molecular modeling. Foods 14:2422. doi: 10.3390/foods14142422 40724243 PMC12295914

[B14] Ghanem H. Cruz C. (2025). Fine-tuning or prompting on LLMs: evaluating knowledge graph construction task. Front. Big Data 8:1505877. doi: 10.3389/fdata.2025.1505877 40636754 PMC12237976

[B15] Ghosh S. Mittal G. (2025). Advancing engineering research through context-aware and knowledge graph–based retrieval-augmented generation. Front. Artif. Intell. 8:1697169. doi: 10.3389/frai.2025.1697169 41346860 PMC12672433

[B16] Gu Y. Dong L. Wei F. Huang M. (2023). “Pre-training to learn in context,” in ACL (Association for Computational Linguistics), 4849–4870. doi: 10.18653/v1/2023.acl-long.267

[B17] Hendrycks D. Burns C. Basart S. Zou A. Mazeika M. Song D. . (2021). “Measuring massive multitask language understanding,” in ICLR (OpenReview.net).

[B18] Hinton G. E. Roweis S. T. (2002). “Stochastic neighbor embedding,” in NIPS (MIT Press), 833–840.

[B19] Huang T. Lee D. H. Sweeney J. Shi J. Steliotes E. Lange M. . (2025). “Foodpuzzle: toward developing large language model agents as autonomous flavor scientists,” in KDD (ACM), 5493–5504. doi: 10.1145/3711896.3737384

[B20] Ji Z. Lee N. Frieske R. Yu T. Su D. Xu Y. . (2023). Survey of hallucination in natural language generation. ACM Comput. Surv. 55, 248, 1–248, 38. doi: 10.1145/3571730

[B21] Jiang L. Jiang J. Wang X. Zhang Y. Zheng B. Liu S. . (2022). Iup-bert: identification of umami peptides based on bert features. Foods 11:3742. doi: 10.3390/foods11223742 36429332 PMC9689418

[B22] Kampars J. Mosans G. Jogi T. Roters F. Vajragupta N. (2025). Llm-supported collaborative ontology design for data and knowledge management platforms. Front. Big Data 8:1676477. doi: 10.3389/fdata.2025.1676477 41312076 PMC12646930

[B23] Kimi-Team (2025). Kimi K2: open agentic intelligence. CoRR, abs/2507.20534.

[B24] Knoedler L. Vogt A. Alfertshofer M. Camacho J. M. Najafali D. Kehrer A. . (2024). The law code of chatGPT and artificial intelligence–how to shield plastic surgeons and reconstructive surgeons against Justitia's sword. Front. Surg. 11:1390684. doi: 10.3389/fsurg.2024.1390684 39132668 PMC11312379

[B25] Li L. He Y. Xu R. Chen B. Han B. Zhao Y. . (2025a). Synchronizing llm-based semantic knowledge bases via secure federated fine-tuning in semantic communication. Front. Artif. Intell. 8:1690950. doi: 10.3389/frai.2025.1690950 41211052 PMC12592048

[B26] Li Y. Shao S. Milling M. Schuller B. W. (2025b). Large language models for depression recognition in spoken language integrating psychological knowledge. Front. Comput. Sci. 7:1629725. doi: 10.3389/fcomp.2025.1629725

[B27] Liu Y. Ding S. Zhou S. Fan W. Tan Q. (2024). Moleculargpt: open large language model (LLM) for few-shot molecular property prediction. CoRR, abs/2406.12950.

[B28] Lun Z. Wu X. Dong J. Wu B. (2025). Deep learning-enhanced spectroscopic technologies for food quality assessment: convergence and emerging frontiers. Foods 14:2350. doi: 10.3390/foods14132350 40647102 PMC12248972

[B29] Moëll B. Farestam F. Beskow J. (2025). Swedish medical LLM benchmark: development and evaluation of a framework for assessing large language models in the Swedish medical domain. Front. Artif. Intell. 8:1557920. doi: 10.3389/frai.2025.1557920 40718621 PMC12290221

[B30] OpenA I. (2025). Gpt-5.1. Technical report, OpenAI.

[B31] Osinomumu I. Reddy U. Doretto G. Adjeroh D. (2025). A survey of machine learning techniques in flavor prediction and analysis. Trends Food Sci. Technol. 166:105339. doi: 10.1016/j.tifs.2025.105339

[B32] Ouyang L. Wu J. Jiang X. Almeida D. Wainwright C. L. Mishkin P. . (2022). “Training language models to follow instructions with human feedback,” in NeurIPS. doi: 10.52202/068431-2011

[B33] Polonioli A. (2025). Moving LLM evaluation forward: lessons from human judgment research. Front. Artif. Intell. 8:1592399. doi: 10.3389/frai.2025.1592399 40495932 PMC12149859

[B34] Qi H. Li X. Zhang C. Zhao T. (2025). Improving drug-drug interaction prediction via in-context learning and judging with large language models. Front. Pharmacol. 16:1589788. doi: 10.3389/fphar.2025.1589788 40529494 PMC12171303

[B35] Qin D. Liang X. Jiao L. Wang R. Zhao Y. Xue W. . (2024). Sequence-activity relationship of angiotensin-converting enzyme inhibitory peptides derived from food proteins, based on a new deep learning model. Foods 13:3550. doi: 10.3390/foods13223550 39593966 PMC11592644

[B36] Radford A. Wu J. Child R. Luan D. Amodei D. Sutskever I. . (2019). Language models are unsupervised multitask learners. OpenAI Blog 1:9.

[B37] Randolph C. Michaleas A. Ricke D. O. (2025). Large language models for closed-library multi-document query, test generation, and evaluation. Front. Artif. Intell. 8:1592013. doi: 10.3389/frai.2025.1592013 40842855 PMC12364804

[B38] Runcie N. T. Deane C. M. Imrie F. (2026). Assessing the chemical intelligence of large language models. J. Chem. Inf. Model. 66, 216–227. doi: 10.1021/acs.jcim.5c02145 41411158 PMC12801315

[B39] Seed B. (2026). Seed1.8 model card: towards generalized real-world agency. arXiv preprint arXiv:2603.20633.

[B40] Van der Maaten L. Hinton G. (2008). Visualizing data using t-sne. J. Mach. Learn. Res. 9, 2579–2605.

[B41] Vilakati S. (2025). Prompt engineering for accurate statistical reasoning with large language models in medical research. Front. Artif. Intell. 8:1658316. doi: 10.3389/frai.2025.1658316 41159127 PMC12554733

[B42] Wei J. Wang X. Schuurmans D. Bosma M. Ichter B. Xia F. . (2022). “Chain-of-thought prompting elicits reasoning in large language models,” in NeurIPS. doi: 10.52202/068431-1800

[B43] Weininger D. (1988). Smiles, a chemical language and information system. 1. Introduction to methodology and encoding rules. J. Chem. Inf. Comput. Sci. 28, 31–36. doi: 10.1021/ci00057a005

[B44] Weininger D. Weininger A. Weininger J. L. (1989). SMILES. 2. Algorithm for generation of unique SMILES notation. J. Chem. Inf. Comput. Sci. 29, 97–101. doi: 10.1021/ci00062a008

[B45] White A. D. Hocky G. M. Gandhi H. A. Ansari M. Cox S. Wellawatte G. P. . (2023). Assessment of chemistry knowledge in large language models that generate code. Dig. Disc. 2, 368–376. doi: 10.1039/D2DD00087C 37065678 PMC10087057

[B46] Yang H. Hu M. Most A. Hawkins W. A. Murray B. Smith S. E. . (2024). Evaluating accuracy and reproducibility of large language model performance on critical care assessments in pharmacy education. Front. Artif. Intell. 7:1514896. doi: 10.3389/frai.2024.1514896 39850846 PMC11754395

[B47] Zhong W. Cui R. Guo Y. Liang Y. Lu S. Wang Y. . (2024). “Agieval: a human-centric benchmark for evaluating foundation models,” in NAACL-HLT (Findings), volume NAACL 2024 of Findings of ACL (Association for Computational Linguistics), 2299–2314. doi: 10.18653/v1/2024.findings-naacl.149

[B48] Zhou P. Tim L. H. Cheng Z. Xie K. Li C. Liu W. . (2025). Enhancing molecular property prediction with knowledge from large language models. CoRR, abs/2509.20664.

[B49] Zhuang J. Shi Y. Hou J. He Y. Ye M. Xu M. . (2025). Reasoning-enhanced large language models for molecular property prediction. CoRR, abs/2510.10248.

